# Effect of Dip Coating Polymer Solutions on Properties of Thermoplastic Cassava Starch

**DOI:** 10.3390/polym11111746

**Published:** 2019-10-24

**Authors:** Kittisak Jantanasakulwong, Nattagarn Homsaard, Phanurot Phengchan, Pornchai Rachtanapun, Noppol Leksawasdi, Yuthana Phimolsiripol, Charin Techapun, Pensak Jantrawut

**Affiliations:** 1 School of Agro-Industry, Faculty of Agro-Industry, Chiang Mai University, Mae-Hea, Mueang, Chiang Mai 50100, Thailand; buzz_buss_@hotmail.com (N.H.); yuingying@hotmail.com (P.P.); Pornchai.r@cmu.ac.th (P.R.); noppol@hotmail.com (N.L.); yuthana.p@cmu.ac.th (Y.P.); charin.t@cmu.ac.th (C.T.); 2 Cluster of research and development of pharmaceutical and natural products innovation for human or animal, Chiang Mai University, Chiang Mai 50200, Thailand; pensak.amuamu@gmail.com; 3 Department of Pharmaceutical Sciences, Faculty of Pharmacy, Chiang Mai University, Chiang Mai 50200, Thailand

**Keywords:** interfacial adhesion, interaction, water resistant, plasticizer bleeding, delaminate

## Abstract

Thermoplastic starch (TPS) was prepared by melt-mixing cassava starch with glycerol. Polyethylene (PE), polyethylene-grafted-maleic anhydride (PE-MAH) and poly(lactic acid) (PLA) solutions at 2% (*w/v*) were used to coat TPS using the dip coating process. The tensile strength of TPS increased with the dip coating solution technique, especially for PLA coating. Swelling index, water-soluble matter and water droplet contact angle confirmed the water resistant improvement of TPS by PE-MAH and the PLA dip coating solution. Plasticizer bleeding was found in uncoated TPS after storage, but not in the coated TPS. Coating TPS with PE-MAH and PLA improved the tensile properties, water resistance and conquered plasticizer bleeding problems in TPS.

## 1. Introduction

Plastics are widely used around the world, such as in food packaging, agricultural, automobile and medical. Although plastics are a more convenient material than others, some plastic compounds cause harm to the health of the users, and also affect the environment due to the long degradation process. 

Bio-materials have been developed to replace petroleum-based polymers by the advantage of degradation ability. Thermoplastic starch (TPS) is a bio-plastic material which is made from starch blending with a plasticizer such as water or glycerol [1]. This thermoplastic material made from natural raw materials is environmentally friendly, and can be decomposed naturally by microorganisms [2]. However, TPS has very poor properties, such as high brittleness, poor water resistance and sensitivity to moisture [3]. The improvement of TPS properties by coating with poly(lactic acid) (PLA) [4], beeswax [5], chitosan [6,7,8], or plasma [9] has been reported. The blending of TPS with other polymers, such as with PLA [5,7,8], nanofibers [10], calcium gluconate [11], polypropylene [12], titanium dioxide [13], poly(butylene adipate-co-terephthalate) [14,15], chitosan [16], polyethylene (PE) [16,17], carboxymethyl cellulose [18], epoxidized natural rubber [19], poly(3-hydroxybutyrate-co-3-hydroxyvalerate) [20] and poly(caprolactone) [21] has been documented. However, the solution for the plasticizer bleeding problem in TPS has not been reported.

Coating technique is an effective and economic methods to improve the properties of materials. Interfacial adhesion between samples and coating materials is the most important factor to develop the properties of it by the coating technique. However, finding the effect of interfacial adhesion between coating materials with bio-materials upon its properties has not been achieved so far. 

The objective of this research was to study the effect of interfacial adhesion between coating materials and TPS to the coated TPS properties. TPS was coated in several polymer solutions including polyethylene (PE), polyethylene-grafted-maleic anhydride (PE-MAH) and poly(lactic acid) (PLA). PE, PE-MAH and PLA were selected due to high tensile strength, high water resistance and the different functional groups of polymers. The mechanical properties, water resistance and plasticizer bleeding of the resulting TPS were investigated.

## 2. Materials and Methods

Cassava starch (molecular weight of 1.34 × 10^8^ g/mol and amylose/amylopectin content of 17%/83%, Dragon Fish brand) was purchased from Tong Chan registered ordinary partnership, Bangkok, Thailand. Glycerol (grade AR) was a product from Quality Reagent Chemical (QREC), Auckland, New Zealand. Polyethylene (Thai-ZEX 1600J, high density polyethylene, melt flow index of 18 g/10 min) was purchased from Polyethylene Public Co., Ltd., Rayong, Thailand. Polyethylene-grafted-maleic anhydride (Fusabond, EMB226-D, backbone of Polyethylene (PE)-LLD and maleic anhydride (MAH) content of 2%) with density of 0.93 g/cm^3^ and a melt flow index of 1.75 g/10 min was gifted by DuPont Co., Ltd. Bangkok, Thailand. Poly(lactic acid) (grade 4032D, D-isomer < 2%, *M*_w_ 218000 g·mol^−1^) was supplied by NatureWorks, Zaandam, Netherland. Toluene (AR grade) and chloroform were purchased from RCI Labscan Asia Co., Ltd., Bangkok, Thailand. 

### 2.1. Sample and Coating Material Preparation

Thermoplastic starch (TPS) was prepared by mixing cassava starch (70 g) and glycerol (30 g) in 500 mL of distilled water for 10 min by an overhead stirrer at a speed of 500 rpm at 80 °C. TPS was dried using a hot air oven at 60 °C for 40 h. The premix sample was subsequently melt-blended using a two-roll mill (Pirom-Olarn Co. Ltd., Bangkok, Thailand, PI-140) at 130 °C for 5 min. The samples were prepared as sheets (thickness of 0.5 mm.) by compression molding (Model 41 22-436, Carver, Inc., Wabash, IN, USA) at 130 °C for 3 min with a pressure of 10,000 Pa. The coating materials were dissolved in specific solvents (Table 1) with 2% *w/v*. Concentration of coating solutions were fixed at 2% *w/v* to study effect of interfacial adhesion to the coated material properties of polyethylene (PE), polyethylene-grafted-maleic anhydride (PE-MAH) and polylactic acid or polylactide (PLA) were selected as coating materials, due to high tensile strength, water resistance and the difference of functional groups. TPS was dipped into the coating material solution, and then put into a hot-air oven at 40 °C for 3 h to evaporate the residual solvent.

### 2.2. Tensile Measurement

The tensile properties of the samples were measured using a tensile tester (Instron universal testing H1KS, Hounsfield Test Equipment, Redhill, UK). The shaped samples were prepared as sheets by compression molding at 130 °C for 3 min with a gauge length, width and thickness of 10, 5 and 0.5 mm, respectively. Tensile samples were dried at 60 °C for 8 h. and observed at a speed of 2 mm/min. 

### 2.3. Swelling Test

The samples were prepared as sheets by compression molding at 130 °C for 3 min with a width × length × thickness of 50 mm × 50 mm × 1 mm. The sample sheets were soaked in water to determine the rate of aquatic fusion in dishes containing 100 mL of distilled water, and stored at 25 ± 2 °C for 24 h. The weight of the samples before and after immersion was observed, and the percentage swelling index (% Swelling Index) was calculated using the following Equation (1):

(1) $$\mathrm{Swelling}\;\mathrm{Index}\;(\%)=\left[\frac{W_{s}-W_{o}}{W_{o}}\right]\times 100$$

where *W*_o_ is weight of the sample before swelling, and *W*_s_ is weight of the sample after swelling.

### 2.4. Solubility Test

The samples were immersed in 100 mL of distilled water for 24 h at 25 °C under stirring (Shaker; Model WNB 22, Memmert, Schwabach, Germany) at 75 rpm, followed by drying the remaining sample in a hot-air oven at 60 °C for 24 h. The weight of the samples before and after stirring in water was measured, and the water solubility was calculated using the following Equation (2):

(2) $$\mathrm{Solubility}\;(\%)=\left[\frac{W_{o}-W_{f}}{W_{o}}\right]\times 100$$

where *W*_o_ is weight of the sample before dissolving, and *W*_f_ is dry-weight of the remained sample after dissolving.

### 2.5. Contact Angle

Drop shape analysis (DSA 30 B, Kruss, Hambrug, Germany) was used to observe the water droplet contact angle. The samples were prepared as sheets by hot-compression at 130 °C for 3 min. The samples were conditioned at 55 ± 5% RH for 40 h. at 23 ± 2 °C. Water was dropped (3 µL; n = 5) onto the surface of the samples, and images of the water droplet angle were taken every min for 1–3 min. 

### 2.6. Measurement of Adhesive Energies

**Figure 1:**
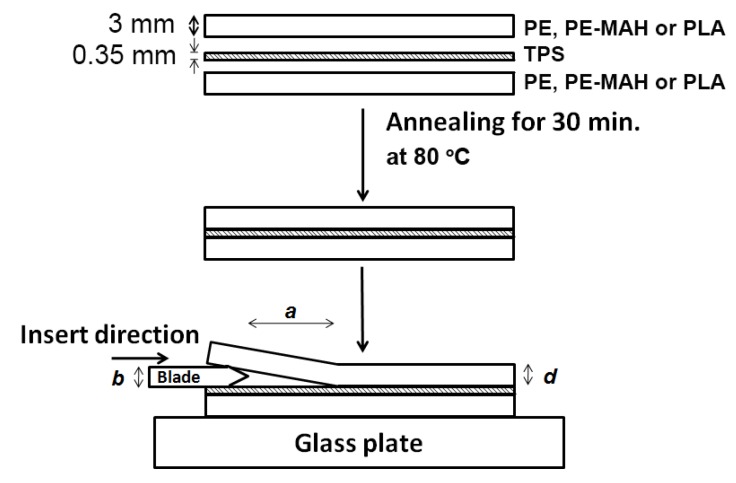
Sample preparation for the asymmetric double-cantilever beam (ADCB) method.

Fracture toughness (*G*_c_) was observed by the asymmetric double-cantilever beam (ADCB) method. The samples were prepared and calculated following the method of [18]. Figure 1 shows the sample preparation for the ADCB test. PE, PE-MAH and PLA sheets were prepared with 3 mm thickness by compression molding. TPS film with the dimension of 5.0 cm × 0.5 cm × 0.3 cm. (length, width and thickness, respectively) was compressed at 130 °C for 3 min. The three-layered specimens of the stacked sheet–film–sheet were annealed at 80 °C for 30 min. The samples were placed on a glass plate and we pushed a razor blade with 0.15 mm of thickness into the sandwich sample 1 cm from the edge. The crack tip was observed by optical microscope. The blade was pushed 0.5 cm and we observed the crack tip, repeating this three times. The *G*_c_ was calculated using the following Equation (3):

(3) $$G_{c}=\frac{3Ed^{3}b^{2}}{8a^{4}{\left[1+\left(0.64d/a\right)\right]}^{4}}$$

where *E*, *a*, *b* and *d* are the sheets (PE, PE-MAH and PLA) Young′s modulus, crack length, thickness of the razor blade and the upper sheet, respectively. The Young′s moduli of PE, PE-MAH and PLA were 455, 210 and 1400 MPa, respectively.

### 2.7. Plasticizer Bleeding Test

The samples were compressed as sheets at 130 °C for 3 min. The samples were conditioned at 55 ± 5% RH and 28 ± 2 °C for two months. An optical microscope was used to observed surface of the sample to study the releasing of glycerol on the sample surface. 

## 3. Results and Discussion

### 3.1. Mechanical Properties

**Figure 2:**
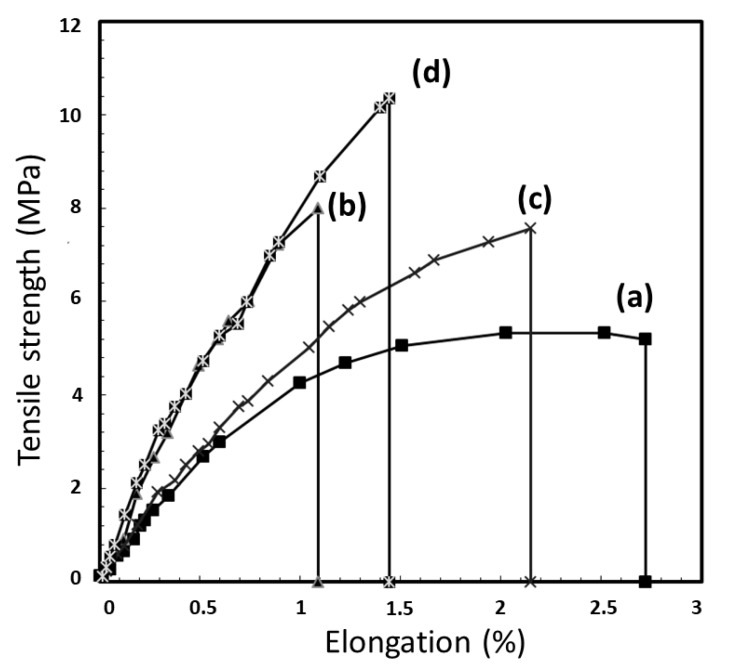
Tensile curves of (**a**) TPS, (**b**) TPS//PE, (**c**) TPS//PE-MAH and (**d**) TPS//PLA.

The tensile strength of TPS and the TPS coated with polymer solutions is presented in Figure 2. TPS showed low tensile strength (5 MPa), Young′s modulus (500 MPa) and elongation at break (2.7%) (Figure 2a). The TPS//PE and the TPS//PE-MAH exhibited increasing tensile strength compared to uncoated TPS, while the TPS//PE presented a higher Young′s modulus (840 MPa) than the TPS//PE-MAH (571 MPa) due to the higher melt flow index of PE (high density polyethylene type) than PE-MAH (low density polyethylene type). The TPS//PLA had the highest tensile strength and Young’s modulus (850 MPa) because of the high mechanical properties of PLA, and the good adhesion of the TPS//PLA coating. The elongation at the break of the coated TPS decreased due to the brittleness of the coating polymers, especially for the PLA coating. It was suggested that the applied strength was distributed through the coating materials and improved the tensile strength of the TPS.

### 3.2. Swelling Test

**Figure 3:**
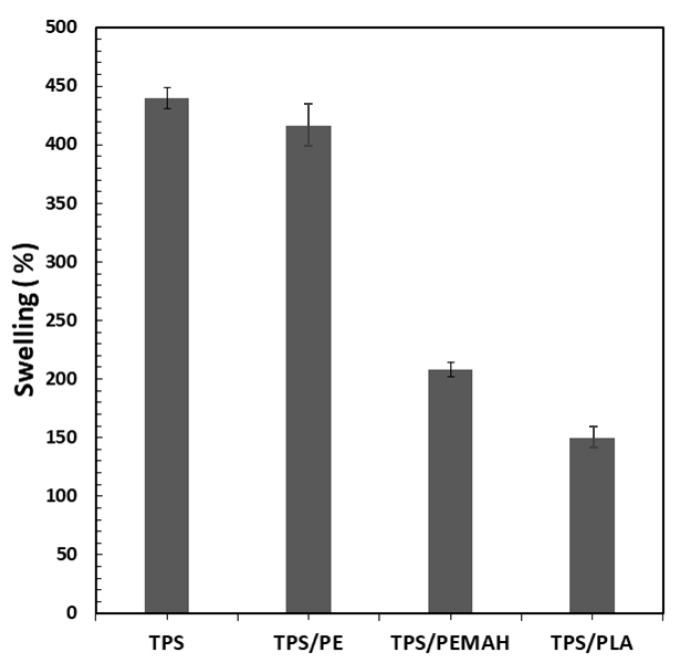
Swelling degree of TPS, TPS//PE, TPS//PE-MAH and TPS//PLA.

The swelling test was used to observe the water resistance of the samples. Uncoated TPS sheets showed the highest swelling degree (440%) (Figure 3). The swelling degree decreased with PE, PE-MAH and PLA coatings, respectively, because of the hydrophobicity of the coating materials. The high swelling degree of the coating PE was due to the poor adhesion of this PE coating on the TPS surface. In the TPS//PE, delamination of the PE layer was observed, and then water penetrated the hydrophilic TPS phase. The low swelling degree of PE-MAH and PLA coating was associated with the hydrophobic nature of both coatings and the good adhesion with the TPS surface.

### 3.3. Solubility Test

**Figure 4:**
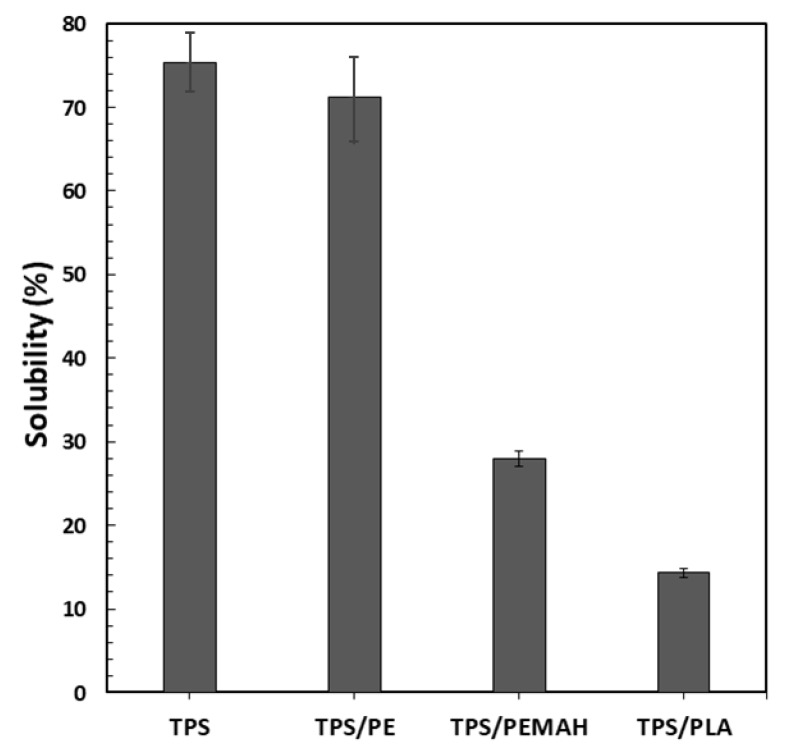
Solubility equilibrium of TPS, TPS//PE, TPS//PE-MAH and TPS//PLA.

Uncoated TPS sheet showed the highest dissolution percentage (75%), and was the most soluble (Figure 4). The TPS//PE and TPS//PE-MAH exhibited decreasing solubility percentages, respectively. The solubility of TPS//PE was close to that of TPS because of delamination of the PE coating layer. Low interfacial adhesion between PE and TPS was expected, thus enhancing the delamination of the PE layer. The PE-MAH coating prevented the water solubility of TPS because of its high interfacial adhesion. The different interfacial adhesion between PE and PE-MAH with TPS suggested a reaction between MAH of PE-MAH with OH of TPS. The TPS//PLA presented a low solubility percentage due to the high compatibility between PLA and TPS [5,7,8]. The intrinsic interaction induced the good interfacial adhesion of TPS with the PLA coating layer, resulting in good water resistance and lower water solubility.

### 3.4. Contact Angle

**Figure 5:**
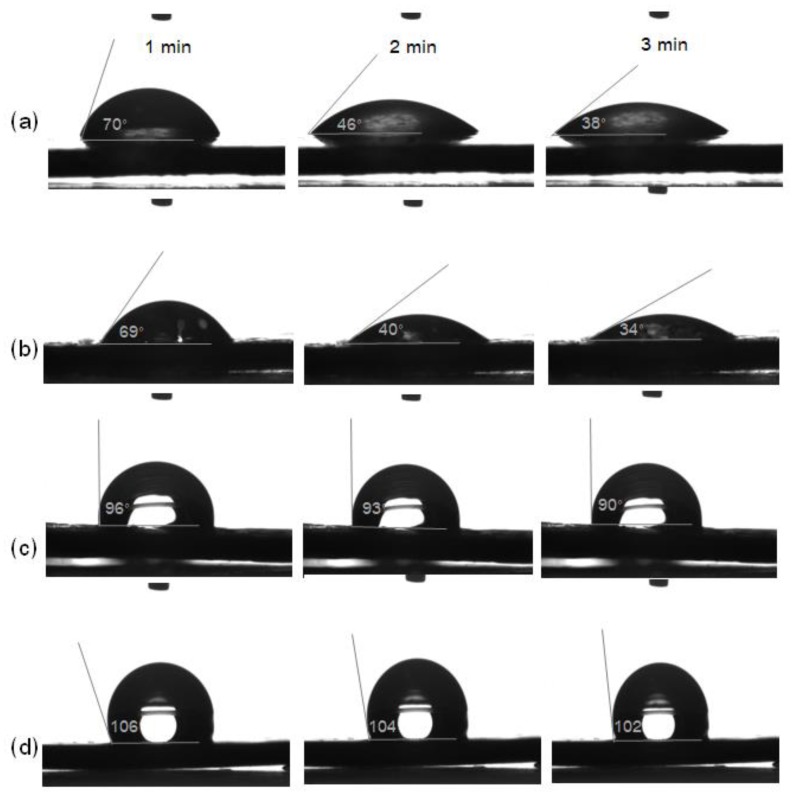
Water droplet contact angle of (**a**) TPS, (**b**) TPS//PE, (**c**) TPS//PE-MAH and (**d**) TPS//PLA at 1–3 min.

The wettability of samples was observed by the water drop contact angle with recording times of 1, 2 and 3 min after dropping the water droplet onto the sample surface. The TPS showed a water contact angle of 70° at 1 min, which decreased to 38° at 3 min (Figure 5a). The decrease in the water contact angle with the recording time indicated the low surface tension of the hydrophilic TPS. The TPS//PE (Figure 5b) also exhibited a similar trend of the water contact angle as TPS (Figure 5a). For TPS//PE-MAH (Figure 5c) and TPS//PLA (Figure 5d) samples, the water contact angle slightly decreased (96–90° and 106–102° at 1–3 min, respectively). Coating the TPS with PE-MAH and PLA resulted in a high water contact angle due to the high surface tension of the coating materials. Based on the results of swelling, solubility and contact angle, it was confirmed that the PE-MAH and PLA coatings improved the water resistance of TPS due to the hydrophobicity of coating materials, the fine coating layer on the TPS surface as a result of dipping in the polymer solution, and the high interfacial adhesion between coating materials with TPS.

### 3.5. Adhesive Energies

The fracture toughness (*G*_c_) between two polymers is an indicator of their adhesion strength. The asymmetric double-cantilever beam (ADCB) method was used to observe *G*_c_. PE showed low adhesion strength with TPS (1.5 J/m^2^), while PE-MAH demonstrated a higher adhesion strength (38.9 J/m^2^) (Table 2). PLA presented the highest adhesion strength with TPS (74.5 J/m^2^). High interfacial adhesion between PE-MAH and TPS suggested a reaction between the MAH of PE-MAH and the –OH of TPS, as previously reported [22], while the high interfacial adhesion between PLA and TPS indicated its high compatibility [5,7,8] and high interaction between the amorphous PLA and TPS surface. The low adhesion strength between TPS and PE caused the delamination of the PE layer on the TPS surface, and presented poor properties, compared with PE-MAH and PLA. This indicated that the high interfacial adhesion of PE-MAH and PLA with TPS induced a fine coating surface by the coating process, which improved the mechanical properties and water resistance of TPS.

### 3.6. Plasticizer Bleeding Test

**Figure 6:**
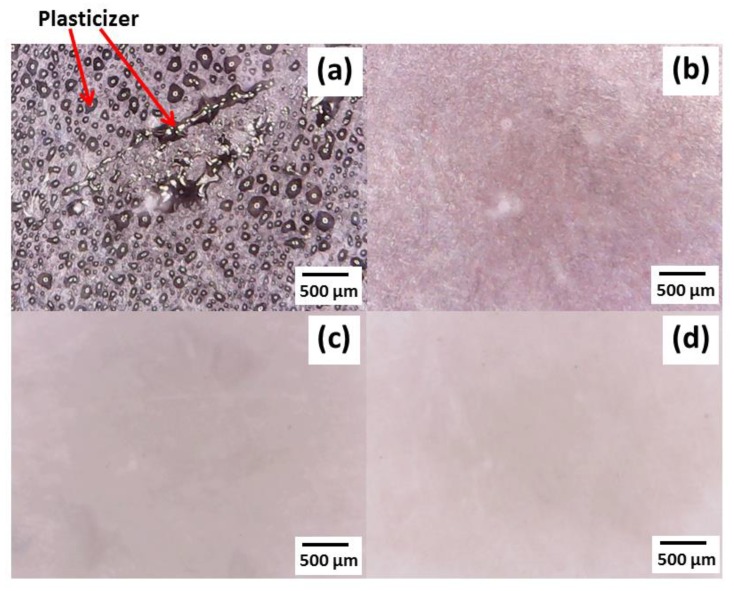
Surface morphology of (**a**) TPS, (**b**) TPS//PE, (**c**) TPS//PE-MAH and (**d**) TPS//PLA and coated TPS by optical microscope after two months of storage.

Plasticizer bleeding is a problem when TPS was stored for a long time. The plasticizer release was observed by optical microscope after two months of storage. Glycerol release onto the uncoated TPS surfaces was observed after two months of storage while the TPS coated with PE, PE-MAH and PLA did not show plasticizer release on the surface. (Figure 6) Plasticizer forms an interaction between –OH groups with starch, and then induces the remaining plasticizer inside the starch.

**Figure 7:**
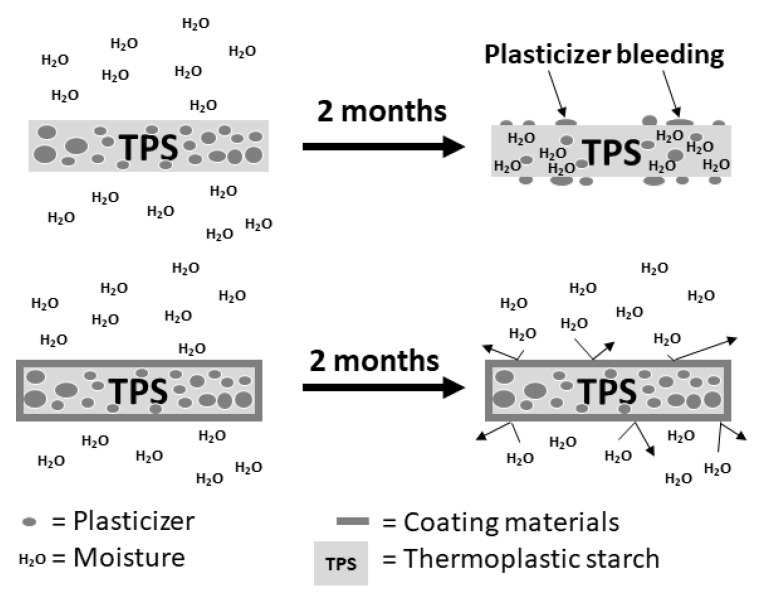
Plasticizer bleeding in TPS and the coated TPS mechanisms.

The general phenomena of plasticizer bleeding in TPS and the coated TPS are shown in Figure 7. The small molecule of water interacted with the –OH groups of starch better than the big molecule of glycerol. When the TPS is kept for a long time in high humidity, the –OH interaction is replaced, and plasticizer is pushed out by moisture. This indicates that the polymer coating prevented the plasticizer from releasing TPS by preventing moisture entering from the outside and trapping plasticizer inside the coated TPS.

## 4. Conclusions

Coating TPS was successfully developed using a dipping process and polymer solutions. The mechanical properties of the coated TPS were improved by coating with polymer solutions due to the high tensile strength of the coating materials and the distribution strength through the coating material layer. Properties of the coating materials were presented via TPS adhesion. The low water resistance of the TPS//PE was due to the low interfacial adhesion between TPS and PE, which induced the delamination of the coating layer. Coating PE-MAH and PLA improved the water resistance of TPS because of its high interfacial adhesion and hydrophobicity. Plasticizer bleeding after the long-term storage of TPS was solved by the high water resistance and the plasticizer trapping of the coating materials. The TPS properties were most improved by PE-MAH and PLA coatings due to the fine coating surface layer, high interfacial adhesion, high tensile strength and hydrophobicity of the coating materials. This coating technique can be applied to hydrophilic materials as a high water resistance packaging or medical application. The properties development of TPS by this coating technique is an effective and economical process to apply for the biopolymers production industry. Tensile strength of the coated TPS was close to polyethylene, which was used to replace this petroleum polymer. In addition, coating TPS with PLA is environmentally friendly, producing a material that can be completely degraded.

## Figures and Tables

**Table 1:** Formulation of thermoplastic starch (TPS) coating.

Sample	Coating Material (2% *w*/*v*)	Solvent	Temperature (°C)
TPS//PE	Polyethylene (PE)	Toluene	70
TPS//PE-MAH	Polyethylene-grafted maleic anhydride (PE-MAH)	Toluene	70
TPS//PLA	Poly(lactic acid) (PLA)	Chloroform	25

**Table 2:** Fracture toughness of two polymers formed by the ADCB method.

Samples	TPS/PE	TPS/PEMAH	TPS/PLA
G_c_ (J/m^2^)	1.5 ± 0.15	38.9 ± 2.14	74.5 ± 4.2
